# Dietary Effect of *Withania somnifera* Root Powder on Growth, Hematobiochemical Parameters, Immunity, and Disease Resistance to *Aeromonas hydrophila* in *Cyprinus carpio*

**DOI:** 10.1155/2024/7321287

**Published:** 2024-06-13

**Authors:** Syed Sikandar Habib, Muhammad Qamar Saeed, Samrah Masud, Osman Sabri Kesbiç, Javed Ahmed Ujan, Cristina Cravana, Salim S. Al-Rejaie, Mohamed Mohany, Francesco Fazio

**Affiliations:** ^1^Department of Zoology, University of Sargodha, Sargodha 40100, Punjab, Pakistan; ^2^Department of Microbiology and Molecular Genetics, Bahauddin Zakariya University Multan, Multan 60800, Punjab, Pakistan; ^3^Institute of Zoology, Bahauddin Zakariya University Multan, Multan 60800, Punjab, Pakistan; ^4^Department of Animal Nutrition and Nutritional Diseases, Kastamonu University Veterinary Faculty, Kastamonu 37150, Türkiye; ^5^Department of Zoology, Shah Abdul Latif University, Khairpur 66020, Sindh, Pakistan; ^6^Department of Animal Sciences, University of Florida, Gainesville 32608, FL, USA; ^7^Department of Veterinary Sciences, University of Messina, Via Palatucci, n. 13, Messina 98168, Italy; ^8^Department of Pharmacology and Toxicology, College of Pharmacy, King Saud University, P.O. Box 55760, Riyadh 11451, Saudi Arabia

## Abstract

This study investigates the influence of *Withania somnifera* root powder (WSRP) on different aspects of common carp (*Cyprinus carpio*), including growth, hematobiochemical parameters, antioxidant status, nonspecific immune response, and resilience to bacterial infections. Over a 60-day period, 180 common carp fingerlings (11.73 ± 0.52 g) were subjected to diets supplemented with increasing WSRP levels (0 (control), 1% (WSRP1), 2.5% (WSRP2.5), and 4% (WSRP4)). Nonspecific immune parameters were evaluated using serum samples collected at intervals of 0, 20, 40, and 60 days. After the feeding trial, the fish underwent experimental challenge with *Aeromonas hydrophila*, and relative percentage survival (RPS) was monitored for 14 days. The findings revealed a considerable (*p*  < 0.05) enhancement in growth performance and a decreased feed conversion ratio (FCR) with increasing WSRP supplementation. Additionally, hematological and biochemical profiles exhibited improvements in groups receiving WSRP-enriched diets. Fish serum antioxidant status showed a significant (*p*  < 0.05) increase, as indicated by increased activities of total antioxidant capacity (TAC), superoxide dismutase (SOD), catalase (CAT), and reduced glutathione (GSH) in WSRP4. WSRP-supplemented diets led to remarkable enhancements in lysozyme activity (*p* = 0.001), phagocytic activity (*p* = 0.002), and RPS (*p* = 0.003), peaking in WSRP4 at day 60. Furthermore, WSRP2.5 and WSRP4 demonstrated a substantial decrease (*p*  > 0.05) in serum glucose and cortisol levels compared to other groups. In conclusion, WSRP proves valuable for enhancing growth, blood parameters, antioxidant balance, immune response, and infection resistance in common carp, especially at 2.5%–4% dietary supplementation levels. In the future, it will be crucial to study the long-term effects of WSRP supplementation on fish health, as well as its potential for large-scale aquaculture and economic viability in the aquafeed industry.

## 1. Introduction

Aquaculture plays a crucial role in providing over 60% of the global food fish supply, and its continual growth appears unavoidable [1]. In the context of intensive aquaculture, where fish solely rely on commercial feeds, the adoption of functional diets becomes imperative to bolster industry sustainability through enhanced growth and heightened immune responses. The assessment of feed additives has been undertaken to strengthen feed utilization, foster growth performance, fortify immune responses, and bolster resistance against diverse pathogens [2, 3]. Natural feed additives, exemplified by medicinal plants, have proven effective in diminishing reliance on chemical treatments or antibiotics. This approach not only reduces metabolic waste production but also enhances fillet quality [4, 5, 6]. Consequently, there is extensive research exploring the potential utilization of medicinal plants in aquaculture.

The expansion of aquaculture has presented a range of challenges, including issues such as sluggish growth, compromised health, and heightened susceptibility to infectious diseases, particularly those of bacterial origin. These diseases, known for their opportunistic nature, pose significant economic threats to both freshwater and marine aquaculture operations [7, 8]. Additionally, fish in intensive production systems face continual exposure to various stressors, including fluctuations in water quality, overcrowding, and temperature variations [9, 10].

The use of conventional medications and vaccines for disease management in aquaculture carries inherent drawbacks [11]. Moreover, the reliance on antibiotics to combat bacterial infections raises concerns about the development of antibiotic resistance and the potential presence of antibiotic residues in fish intended for human consumption [12, 13]. Hence, the need arises to explore safe and cost-effective alternatives to traditional disease control methods, aligning with the principles of environmentally friendly aquaculture.

In recent years, advancements in fish nutrition have led to the development of specialized feeds and innovative, well-balanced commercial diets. These breakthroughs play a pivotal role in achieving optimal growth and producing high-quality, healthy fish [14, 15]. Efforts have been directed toward integrating aromatic and medicinal herbs into feed formulations, introducing novel strategies to mitigate disease risks and enhance the immune system, particularly when fish are exposed to stressors such as poor water quality, transportation, and rough handling in aquaculture environments [16, 17]. Among these, the medicinal plant *Withania somnifera* is native to India and Pakistan and acknowledged for its myriad medicinal properties. *W. somnifera* (L. Dunal) is a member of the Solanaceae family. Studies indicate that *W. somnifera* exhibits a range of beneficial properties, including anti-inflammatory, antitumor, antistress, antioxidant, immunomodulatory, hemopoietic, and rejuvenating effects [18, 19, 20]. The remarkable findings of *W. somnifera* include a study where feed incorporating *W. somnifera* plant extract at a dosage of 200 mg/kg significantly decreased mortalities in juvenile greasy grouper (*Epinephelus tauvina*) facing *Vibrio harveyi* infection [21]. Additionally, postlarvae of *Penaeus monodon*, when fed with *W. somnifera* herbal medicinal diets, exhibited enhanced stress resistance [22]. Numerous studies have been undertaken to assess the effects of *W. somnifera* across various animal species. However, research on the impact of *W. somnifera* on fishes remains limited. To our knowledge, no study has yet examined the dietary effects of *W. somnifera* on *Cyprinus carpio*. This research sets out to bridge existing knowledge gaps by systematically evaluating the impact of *W. somnifera* root powder (WSRP) on the hematology and biochemical profiles of common carp (*C. carpio*). Additionally, the study aims to elucidate how WSRP influences the antioxidant status, nonspecific immune response, and the overall resistance of common carp to *Aeromonas hydrophila* infection.

## 2. Materials and Methods

### 2.1. Plant Preparation

The specimens of *W. somnifera* were bought from a market in the Kohat district of Khyber Pakhtunkhwa, Pakistan. The specimens underwent examination by plant taxonomists at the Department of Botany, University of Peshawar, Pakistan. The plant roots were delicately separated, accurately washed and cleaned with distilled water, and then carefully dried in a shaded, dust-free environment [23]. The dried plant roots were ground into powder using an electric blender, and the resulting powder was stored in airtight containers at room temperature for further use. The phytochemical compounds in the root of *W. somnifera* were confirmed through gas chromatography and mass spectrometry (GCMS) analysis (Tables 1 and 2). The proximate composition of the examined feed was assessed in accordance with the AOAC (2000) protocol.

### 2.2. Conditions for Fish Rearing and Study Design

A total of 180 healthy common carp, *C. carpio* fingerlings, was sourced from a local farm in the Dera Ismail Khan District of Pakistan and transported in adequately aerated containers to the laboratory; the specimens were immersed in a 0.05% KMnO_4_ solution for 5 min to address potential dermal infections, if present. Afterward, a 2-week acclimation period was provided before initiating the experiment. Throughout the acclimation period, the common carp were supplied with the basal diet (Supreme Feeds, Pakistan) and subjected to a 12/12 light–dark cycle. At the same time, vigilant monitoring was maintained to detect any signs of diseases or instances of mortality. In accordance with the standards set by CCoA [24], a regular assessment of the health status of the fish was carried out before the beginning of the experiment. Subsequently, the weights of each individual were documented, revealing an average initial weight of 11.73 ± 0.52 g at the commencement of the experiment. Afterward, the fish were randomly distributed into four groups, each consisting of three replicates in fiberglass tanks (80 L each). Each group included 45 fish, organized into three replicates with 15 fish per replicate. Continuous aeration systems were installed in the tanks.

The experimental groups consisted of four basal diets supplemented with varying doses of *W. somnifera* root powder (WSRP) 0, 1%, 2.5%, and 4% diet (designated as WSRP0, WSRP1, WSRP2.5, and WSRP4, respectively) throughout a 60-day feeding period. The dietary components underwent mechanical blending, yielding pellets with a diameter of 1.5 mm using a pellet machine. Following preparation, the diets were subjected to a 24-hr air-drying process at room temperature and subsequently stored at 4°C until use.  Table 3 illustrates the detailed composition of both the basal and experimental diets, offering a comprehensive insight into the specific ingredients utilized. The fish were fed the experimental diets, amounting to 3% of their total biomass, twice daily (9:00 a.m. and 3:00 p.m.). Every 2 weeks, the feed was modified according to the weight of the fish.

Furthermore, any uneaten feed was collected, dried, and subtracted from the initially supplied amount to ensure an accurate calculation of feed intake. To foster an environment conducive to growth and survival throughout the experiment, we systematically observed and regulated water temperature, dissolved oxygen, and pH levels on a daily basis [25]. The temperature of the water fluctuated within the range of 26.16–26.78°C, while the levels of dissolved oxygen ranged from 6.2 to 6.8 mg/L. The pH levels were recorded between 7.2 and 7.6, and the concentration of ammonia varied from 0.22 to 0.25 mg/L. All the parameters were within the permissible thresholds for fish aquaculture, as specified by Boyd and Tucker [26]. On a biweekly basis, a 25% exchange of tank water, coupled with the removal of accumulated fish excrement, was carried out and replaced with fresh, aerated water.

### 2.3. Growth Performance

Biweekly, the fish underwent sampling to assess their growth performance, employing a precise weight balance. The evaluation of growth performance was done [27] using the provided formulas as follows:

(1) $$\begin{array}{c} \text{Weight gain }\left(\text{WG}\right)=\text{Final body weight }\left(g\right)-\text{Initial body weight }\left(g\right), \end{array}$$

(2) $$\begin{array}{c} \text{Feed intake }\left(\text{FI}\right)=\frac{\text{Feed consumed}}{\text{Number of surviving fish}}, \end{array}$$

(3) $$\begin{array}{c} \text{Specific growth rate }\left(\text{SGR}\right)=\left[\frac{\left(\text{Loge final weight}-\text{Loge initial weight}\right)}{\text{Number of days}}\right]\times 100, \end{array}$$

(4) $$\begin{array}{c} \text{Feed conversion ratio }\left(\text{FCR}\right)=\frac{\text{Feed given }\left(\text{Dry weight}\right)}{\text{Body WG }\left(\text{Wet weight}\right)}, \end{array}$$

(5) $$\begin{array}{c} \text{Survival rate }\left(\text{SR}\right)=\frac{\text{Total number of fishes harvested}}{\text{Total number of fishes stocked}}\times 100. \end{array}$$

### 2.4. Blood Collection

At the termination of the experiment, three fish were randomly chosen from each tank, constituting a group of nine fish, and then anesthetized using clove oil at a concentration of 80 mg L^−1^ [28]. Afterward, blood was drawn from the caudal vessels and collected in vials containing heparin, facilitating the evaluation of hematological parameters. Sterile syringes, devoid of anticoagulants, were employed to collect additional blood samples. Then, these samples underwent centrifugation at 5,000 rpm for 15 min to isolate serum, which was then stored in a refrigerator at −20°C for further use. The biochemical and immunological analyses were performed using the obtained serum samples.

### 2.5. Hematological and Biochemical Indices

The blood parameters analyzed included red blood cell (RBC) counts and white blood cell (WBC) counts, utilizing a Neubauer hemocytometer [29]. Hemoglobin (Hb) concentration was assessed employing the cyanmethemoglobin method, which entailed measuring optical density at 540 nm with the use of spectrophotometry [30]. Hematocrit (Hct) levels were determined by centrifuging microhematocrit tubes at 3,000 rpm for 5 min. For the determination of differential leukocytes, blood smears were meticulously prepared on glass slides and subjected to staining using the May–Grünwald Giemsa process [29]. Spectrophotometric analysis was utilized to examine serum albumin (ALB) at a wavelength of 630 nm and total proteins (TP) at a wavelength of 540 nm, following the methodologies outlined by Reinhold [31] for ALB and Henry [32] for TP. Additionally, serum globulins (GLOB) were quantified using the procedure described by Coles [33]. Serum glucose (GLU) levels were measured with a colorimetric diagnostic kit from Siemens Healthineers, Germany, at a wavelength of 500 nm. The evaluation of serum cortisol (CORT) levels followed the methodology outlined by Tunn et al. [34].

### 2.6. Antioxidant Status

The antioxidant activities in the serum were evaluated using colorimetric commercial kits (Shanghai Kehua Bio-Engineering Co., Ltd.). Serum total antioxidant capacity (TAC) was assessed following the method of Koracevic et al. [35] at 450 nm. Catalase (CAT) and superoxide dismutase (SOD) activities were determined following the procedures outlined by Aebi [36] and Nishikimi et al. [37], respectively, at wavelengths of 510 and 560 nm. The quantification of glutathione dehydrogenase (GSH) through colorimetric analysis was conducted in accordance with the protocol detailed by Beutler [38] at 340 nm.

### 2.7. Immunological Parameters

#### 2.7.1. Serum Lysozyme Activity

The activity of serum lysozyme was assessed employing the turbidimetric method described by Koskela et al. [39]. The highest level of activity was observed in a 0.05 M phosphate buffer (pH = 6.0), wherein the substrate, lyophilized *Micrococcus lysodeikticus*, was suspended at a concentration of 3.0 mg/mL. The experiments were carried out at a temperature of 20°C, and the absorbance at 530 nm was tracked over a time span ranging from 0.5 to 4.5 min, utilizing a spectrophotometer. A unit of lysozyme activity was determined as the amount of enzyme causing a decrease in absorbance by 0.001 per minute.

#### 2.7.2. Phagocytic Activity

Phagocytic activity was conducted using a modified approach inspired by Anderson and Siwicki [40] method. Employing aseptic techniques, blood was aseptically drawn from the fish. Subsequently, 0.1 mL of the blood cell suspension was introduced into a 96-well microtiter plate. To this, 0.1 mL of *Staphylococcus aureus* (1 × 10^7^ cells), suspended in a phosphate-buffered saline (PBS) solution at a 1 : 1 ratio (10^7^ bacteria: PBS), was added and thoroughly mixed. The plate was then incubated for 20 min at room temperature. Following incubation, a smear of the treated suspension was created on a glass slide and allowed to air dry. Subsequently, the prepared smear was fixed with 95% ethyl alcohol for 5 min, followed by air drying. It was then stained with Giemsa (7%) for a duration of 10 min. The stained smear was examined under a microscope to enumerate the cells, typically 100, that were engaged in the process of engulfing bacteria. The calculation for phagocytic activity (PA) was determined using the formula PA = (Number of phagocytizing cells/Number of total cells) × 100. The outcomes were conveyed as a percentage (%).

### 2.8. Challenge Test

To evaluate the resilience of common carp against *A. hydrophila* bacteria, sourced from the Microbiology Department at Punjab University, Pakistan, a total of 15 fish per treatment were randomly chosen—five from each tank. These selected fish were collectively housed in individual tanks corresponding to their respective treatments. Over a trial period of 60 days, involving feeding and periodic blood sampling, each fish received an intraperitoneal injection of 0.1 mL. This injection contained a suspension of *A. hydrophila* at a concentration of “1.5 × 10^6^ CFU mL^−1^” in a phosphate-buffered solution. Careful observation of the fish for any signs of illness or unusual behavior was conducted postinjection. In the event of fish mortality, a gentle removal process was employed to minimize stress on the remaining fish in the tanks. Survival rates across all treatment groups were closely monitored during the 14-day postinfection period. Throughout the postchallenge phase, the fish were provided with experimental diets and were maintained under identical rearing conditions established during the initial feeding period. This comprehensive approach ensured a thorough assessment of the fish resilience to *A. hydrophila*, with specific attention given to survival rates and behavioral changes during the postinfection period.

### 2.9. Relative Percentage Survival (RPS)

Using the provided formula, we calculated the relative percentage survival (RPS) at the conclusion of the 14-day postinfection period, employing the methodology outlined by Amend [41]:

(6) $$\begin{array}{c} \text{RPS}\%=\frac{\text{No. of live fish after challange test}}{\text{No. of fish injected with bacteria}}\times 100 \end{array}$$

### 2.10. Statistical Analysis

Statistical analysis was conducted through a one-way analysis of variance (ANOVA) with SPSS version 28.0. To discern differences between groups, Tukey's multiple comparison post hoc test was applied. The predetermined level of statistical significance was set at *p*  < 0.05.

## 3. Results

### 3.1. Growth Performance of Common Carp

Table 4 presents the outcomes of the growth performance and survival rates of common carp when subjected to a diet supplemented with WSRP. A remarkable influence was evident across all parameters related to growth. The inclusion of WSRP4 in the diets resulted in a significant improvement in WG (*p* = 0.007), SGR (*p* = 0.02), and PER (*p* = 0.003) compared to WSRP1 and WSRP0, with no significant difference from WSRP2.5. The FI was significantly higher (*p* = 0.01) in WSRP4 than in other groups. In terms of FCR, the WSRP4 group exhibited a marked reduction (*p* = 0.001), outperforming WSRP1 and WSRP0. However, there was no significant difference in FCR between WSRP2.5 and WSRP4. Importantly, the survival rate of the fish remained consistently high at 100%, and no significant differences were observed among the different groups throughout the feeding period. Furthermore, the overall health and activity levels of the fish in all test groups were maintained, affirming the positive impact of WSRP supplementation without any adverse effects during the feeding period.

### 3.2. Hematological and Biochemical Parameters of Common Carp

The hematological indices of common carp, outlined in Table 5, reflect the impact of a diet enriched with varying concentrations of WSRP. A significant enhancement in hematological indices was observed in fish fed with WSRP-fortified diets compared to the control group. The augmentation in hematological parameters followed a dose-dependent pattern, revealing a substantial rise with increasing WSRP concentrations, as indicated by the results achieved by the WSRP4 group, closely followed by the WSRP2.5 group. Significantly higher results for RBCs (*p* = 0.03), Hct (*p* = 0.01), lymphocytes (*p* = 0.002), and heterophils (*p* = 0.02) were recorded in fish in the WSRP4 group compared to WSRP1 and WSRP0. Moreover, Hb (*p* = 0.003), WBCs (*p* = 0.001), eosinophils (*p* = 0.001), and monocytes (*p* = 0.01) were considerably higher in the WSRP4 compared to the other groups.

Table 6 presents the serum biochemical indices of common carp, specifically focusing on the impact of an experimental diet containing different concentrations of WSRP. A significant improvement (*p* = 0.006) in the levels of ALB was observed in the WSRP4 group compared to the other groups. Additionally, there was a significant increase in TP (*p* = 0.002) and GLOB (*p* = 0.01) levels in fish fed the WSRP4 and WSRP2.5 diets compared to the WSRP1 and control groups. Conversely, the serum levels of GLU and CORT exhibited a marked reduction (*p* = 0.01 and *p* = 0.02, respectively) in the WSRP4 group compared to the WSRP1 and control groups. However, there was no significant difference in GLU and CORT values between the WSRP4 and WSRP2.5 groups.

### 3.3. Serum Antioxidant Status of Common Carp

The results outlined in Table 7 elucidate the impact of a diet fortified with WSRP on the serum antioxidant enzyme activities in common carp. The investigation revealed significant improvements in the concentrations of key antioxidant enzymes, including TAC (*p* = 0.003), CAT (*p* = 0.002), SOD (*p* = 0.002), and GSH (*p* = 0.001) in the WSRP4 group compared to the other experimental groups and the control.

### 3.4. Immunological Parameters

**Figure 1:**
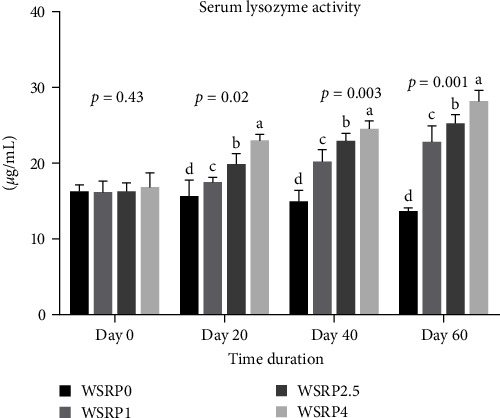
Effects of various concentrations of *W. somnifera* root powder (WSRP) on serum lysozyme activity of *C. carpio*. Different letters indicate significant difference among the groups.

The lysozyme activity levels exhibited an upward trend from the 20th to the 60th day of sampling across all groups (WSRP1, WSRP2.5, and WSRP4) of fish that were fed a diet containing WSRP (Figure 1). Starting from day 20 and continuing through day 40 and day 60 of sampling, there was a notable increase in lysozyme activity. WSRP4 showed significant enhancement on days 20 (*p* = 0.02), 40 (*p* = 0.003), and 60 (*p* = 0.001) compared to the other groups.

**Figure 2:**
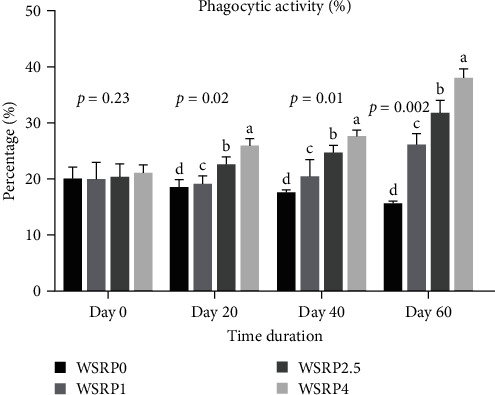
Effects of various concentrations of *W. somnifera* root powder (WSRP) on phagocytic activity of *C. carpio*. Different letters indicate significant difference among the groups.

**Figure 3:**
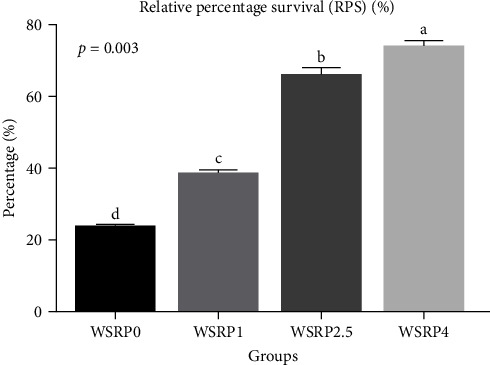
Effects of various concentrations of *W. somnifera* root powder (WSRP) on relative percentage survival of *C. carpio*. Different letters indicate significant difference among the groups.

The phagocytic activity in the WSRP4 group, and subsequently in the WSRP2.5 group, showed an increasing trend from day 20 to day 60 of the sampling period (Figure 2). Notably, the phagocytic activity in WSRP4 was significantly higher on days 20 (*p* = 0.02), 40 (*p* = 0.01), and 60 (*p* = 0.002). The highest RPS was observed in the WSRP4 group and was significantly higher (*p* = 0.003) compared to the control group, followed by the WSRP2.5 and WSRP1 groups (Figure 3).

## 4. Discussion

Aquaculture stands as a promising avenue for providing a sustainable and economical supply of nutritious protein for human consumption, thereby contributing to the enhancement of human health [42]. However, the persistent issue of disease outbreaks presents a formidable obstacle to realizing sustainable production through advanced intensification techniques. A variety of environmentally sound and secure compounds are currently being explored in the field of aquaculture with the goal of regulating immune responses, enhancing growth performance, improving digestive activity, and preventing diseases in fish and other species [43, 44]. Therefore, our investigation aims to examine the potential of WSRP in enhancing the well-being, immune responses, and growth performance of common carp. The outcomes of this study revealed the successful improvement of various health indicators in common carp through the dietary incorporation of WSRP. Particularly significant results emerged from the WSRP4 group, displaying a considerable increase in weight gain (%), SGR, FCR, and PER when compared to those subjected to the control diet. These observations are consistent with the results reported by Srivastava et al. [45], who investigated the influence of WSRP on the growth rate of *Labeo rohita*. As far as our knowledge extends, there is no previous research examining the impact of WSRP on common carp. A noticeable gap exists in studies concentrating on *W. somnifera* as a dietary component for enhancing the health and growth of fish. The adoption of plant-based diets in aquaculture has garnered substantial attention and recognition. Herbs and plants, renowned for their abundance of bioactive compounds, have the potential to elevate the general well-being, growth, and immune reactivity of fish [46]. Such natural supplements normally comprise antioxidants, antimicrobials, and anti-inflammatory agents, fostering resilience against diseases and enhancing the overall vigor of aquatic species [47, 48]. A study conducted by Fazio et al. [6] demonstrated that the fruit extract of *W. coagulans* contributes to increased weight gain, SGR, and better FCR in *L. rohita*. SGR is an important indicator that demonstrates the efficiency and success of a fish culture system. A higher SGR suggests better growth performance, which is often a desirable outcome in aquaculture for achieving larger and more marketable fish in a shorter period [49]. A lower FCR indicates greater efficiency in converting the provided feed into fish biomass [50]. In a fish culture system, achieving a lower FCR is typically desirable because it means that less feed is required to produce a unit of weight gain in the fish. This efficiency is beneficial for both economic and environmental reasons [51]. In our study, higher weight gain, SGR, and lower FCR were recorded in WSRP4, followed by WSRP2.5, compared to the control. In multiple investigations, plant-based diets have demonstrated significant effects on the growth of *C. carpio*. For instance, Yousefi et al. [3] noted increased weight gain, SGR, and decreased FCR in fish that were provided with a supplemented diet containing *Origanum majorana* extract. Similarly, Ghafarifarsani et al. [52] reported improved growth performance in fish-fed diets supplemented with *Origanum vulgare*, *Malvae sylvestris*, and *Allium hirtifolium* boss. In another study conducted by Ghafarifarsani et al. [53], it was observed that *C. carpio* showed enhanced weight gain, SGR, and lower FCR when provided with a diet fortified with savory (*Satureja hortensis*) essential oil. PER serves as a valuable metric for determining the nutritional quality of the feed and evaluating how efficiently the dietary protein fosters fish growth. Enhanced PER values indicate more effective utilization of protein for growth, signifying a well-balanced diet [54]. Furthermore, the optimal intake of feed plays a pivotal role in the overall success of aquaculture operations, fostering growth, meeting nutritional needs, and supporting the health of the fish population [55]. Additionally, increased levels of protein, carbohydrates, essential oils, fiber, vitamins, and minerals contribute to an improved nutritional profile within the diet [6].

The evaluation of fish health and physiological status involves scrutinizing hematobiochemical parameters [56]. Our current investigation confirms that common carp, when fed with WSRP, demonstrated a noteworthy increase in all blood indices, particularly with diets containing 4% of root powder. *W. somnifera* includes a diverse array of phytochemicals, including flavonoids, alkaloids, phenolics, steroids, terpenoids, glycosides, saponins, proteins, and carbohydrates [57]. Our study aligns with the findings of a single investigation on *W. somnifera* conducted by Laltlanmawia et al. [2]. In their research, they observed improved hematological parameters in *L. rohita* cultured for 60 days. Other studies also revealed enhanced hematological parameters in *C. carpio*, such as Baba et al. [58] used a fortified diet with *Avena sativa* extract, and Kavitha et al. [59] provided a supplemented diet with seed extract of *Moringa oleifera*. Increased levels of RBCs, Hb, and HCT in fish are commonly associated with an enhanced ability to transport oxygen in the bloodstream [50]. This phenomenon implies improved oxygen delivery to tissues, a critical factor for diverse physiological functions such as metabolism and overall health. Elevated WBC counts and positive alterations in the differential leukocyte count in fish, attributed to better diet, have the potential to boost immune system functionality and overall health [60]. A nutritionally rich diet may play a role in strengthening the immune response in fish, aiding in their resistance to infections [13]. In the present study, fish fed a fortified diet based on WSRP exhibited significant improvements in both WBC count and differential leukocyte levels.

In this investigation, common carp exhibited a significant increase in albumin, total protein, and globulin levels when nourished with 4% of WSRP, followed by 2.5% and 1%, and then the control group. These results align with the findings made by Sokooti et al. [61], who documented elevated levels of these parameters in *C. carpio* when subjected to a diet incorporating olive leaf extract. Albumin, a pivotal protein essential for diverse physiological functions such as upholding osmotic equilibrium, facilitating the transport of hormones and nutrients, and playing a role in the overall protein composition of fish, has been highlighted in studies by Susilowati et al. [62] and Habib et al. [54]. Consequently, an increased concentration of albumin is commonly linked to a nutritionally rich diet. Additionally, the increased concentrations of total protein and globulin observed in the present investigation align with the results reported by Mohammadi et al. [63], who administered *Phoenix dactylifera* seed extract to *C. carpio*. The utilization of WSRP demonstrates the capacity to boost defensive proteins, consequently triggering an activation of the immune system. Higher levels of blood proteins, particularly globulins, act as a favorable signal, indicating enhanced liver function and a heightened innate immune response [64, 65]. Primary indicators of stress in fish encompass variations in serum glucose and cortisol levels, responsive to shifts in environmental conditions or dietary influences, as emphasized by Habib et al. [54]. Under normal circumstances, cortisol assumes a regulatory function in diverse physiological processes within fish, facilitating prompt adjustments in response to stress [66]. Cortisol acts as a stimulator for various elements of intermediary energy metabolism, amplifying oxygen absorption, fostering gluconeogenesis, and impeding glycogen synthesis [67]. In the current investigation, the introduction of WSRP into the dietary regimen resulted in a significant decrease in both blood glucose and cortisol levels. The presence of abundant flavonoids and essential minerals in *W. somnifera* plays a pivotal role in the regulation of glucose uptake and lipid metabolism [68]. Parallel findings were observed in other studies, where *C. carpio* exhibited reduced cortisol and glucose levels when fed a diet enriched with *Myristica fragrans* extract [69] or supplemented with *Zingiber officinale* [70].

Oxidative stress poses a risk of harming crucial biological components like proteins and DNA. In response, the body activates a protective mechanism to counteract and minimize oxidative damage to its tissues [71, 72]. CAT and SOD are enzymatic antioxidants that effectively neutralize reactive oxygen free radicals, while glutathione, a nonenzymatic antioxidant, mitigates these radicals through enzymatic reactions. In the current study, a significant increase in the activities of TAC, CAT, SOD, and GSH in fish serum, compared to control levels, was observed when the diet was enriched with WSRP. This phenomenon can be attributed to the presence of bioactive compounds in *W. somnifera*, including flavonoids, steroids, terpenoids, glycosides, alkaloids, polyphenols, and vitamins. These compounds are renowned for their antioxidant and anti-inflammatory properties [57]. Other studies have consistently affirmed analogous results, indicating an augmented antioxidant status in fish when exposed to a plant-based fortified diet. Remarkable instances include the study conducted by [73], which observed increased antioxidant levels in *C. carpio* following the inclusion of *Artemisia absinthium* extract. Similarly, findings from the research by Rashidian et al. [69] revealed comparable outcomes in *C. carpio* through the application of *M. fragrans* extract. Moreover, Ghafarifarsani et al. [74] showed higher CAT and SOD activities in rainbow trout (*Oncorhynchus mykiss*) fed on 1.5% and 2% of *Calendula officinalis* powder.

The challenge test serves as a widely adopted standard assay for assessing the overall health of the immune system [75]. The increased resilience of fish to pathogenic microorganisms stands out as a crucial indicator of the efficacy of immunostimulants [13]. In line with the findings of this investigation, the introduction of WSRP into the diet demonstrated a protective influence against *A. hydrophila* infection in the fish. This potential connection may be ascribed to elevated levels of nonspecific immune parameters, including higher lysozyme levels, phagocytic activity, and enhanced antioxidant enzyme activities. Furthermore, the observed reductions in glucose and cortisol levels likely contributed to reinforcing the fish defense against infection. Notably, *W. somnifera* has been acknowledged for its exceptional pharmacological attributes, encompassing antibacterial capabilities among its diverse range of medicinal properties [76]. For the 20th to the 60th day of sampling, there was a consistent upward trend in both lysozyme and phagocytic activity. Notably, the most substantial increase was observed in the WSRP4 group, followed closely by the WSRP2.5 group in this study. These results align with the research conducted by Sharma et al. [23], which investigated the immunomodulatory effects of WSRP on *L. rohita*, and Trivedi et al. [77], who explored the use of dietary supplementation with *Asparagus racemosus* and *W. somnifera* in *Channa punctatus*. In the *A. hydrophila* challenge test, the treatment group (WSRP4) exhibited the highest percentage of survival, followed closely by the group (WSRP2.5) compared to the control group. These results are in concordance with a previous study conducted on *Oreochromis mossambicus*, which is a diet containing *Ocimum sanctum* [78], and a study on common carp, which incorporated a diet supplemented with *Lactobacillus helveticus* and gum Arabic [79].

Although this study provides useful information on the usage of WSRP as feed additives, it lacks information on potential interactions between the supplemented WSRP and other components of the experimental diets. It does not address whether any interactions occurred between the WSRP and other dietary ingredients that could have influenced the observed outcomes on growth performance, hematological and biochemical indices, antioxidant status, and immunological parameters. Additionally, it does not provide information on the long-term effects of WSRP supplementation. Future studies should focus on conducting long-term investigations to understand the sustainability and effectiveness of dietary interventions in aquaculture.

## 5. Conclusion

In summary, the inclusion of WSRP in the diet of common carp at a concentration of 4% has resulted in favorable outcomes, positively impacting various aspects of their health and performance. The observed enhancements in growth, blood parameters, antioxidant status, and immunity indicate that WSRP holds promise in enhancing the overall well-being of common carp, particularly in fortifying their resistance against bacterial challenges, such as those presented by *A. hydrophila*. These results highlight the potential of *W. somnifera* as a beneficial dietary supplement for promoting the robust health and immune response of common carp in aquaculture environments. Further investigation into the underlying mechanisms of these positive effects could contribute to optimizing the utilization of *W. somnifera* in aquaculture practices.

## Figures and Tables

**Table 1:** Phytochemical compounds in *W. somnifera* root using GCMS.

Compound	*W. somnifera* root
Alcoholic compound	+
Aldehyde	+
Alkaloid	+
Alkene compound	+
Ether compound	+
Fatty acid	+
Fatty acid ester	+
Hydrocarbon	+
Iodine compound	−
Ketone compound	+
Lauric acid	+
Myristic acid	+
Nitrogen compound	+
Oleic acid	+
Oxirane compound	−
Palmitic acid	−
Phenolic compound	+
Plasticizer compound	−
Sugar compound	+

Detectable compounds (+) and nondetectable compound (−).

**Table 2:** Identified phytochemical compounds in *W. coagulans* root using GCMS.

Compound name (chemical formula)	Percentage (%)	Retention time
Phenylethyl alcohol (C_8_H_10_O)	1.30	5.75
1-Tridecyne (C_13_H_24_)	1.45	20.43
Propane, 1,1-diethoxy-2-methyl (C_8_H_18_O_2_)	0.36	2.71
3-Hexenoic acid, butyl ester (C_10_H_18_O_2_)	0.97	6.68
Oleic acid (C_18_H_34_O_2_)	1.30	20.55
2,3,4,5-Tetrahydropyrid azine (C_4_H_8_N_2_)	11.07	3.17
8-Azabicyclo [3.2.1]octan-3-ol, 8-methyl-, endo-(C_8_H_15_NO)	0.78	7.38
9-Octadecenal (C_18_H_34_O)	0.84	20.28
Butane, 1,1-diethoxy-2-methyl- (C_9_H_20_O_2_)	11.60	3.67
Sucrose (C_12_H_22_O_11_)	0.47	10.02
Tetradecanoic acid (C_14_H_28_O_2_)	0.81	14.32
1,E-11 Z-13-Octadectriene (C_18_H_32_)	11.60	20.15
2-Nonanone (C_9_H_18_O)	0.84	4.83
Amyl nitrite (C_5_H_11_NO_2_)	23.64	10.72
Decanoic acid, ethyl ester (C_12_H_24_O_2_)	11.07	17.76
Propane, 1,1,3- triethyoxy (C_9_H_20_O_3_)	1.45	5.07
Dodecanoic acid (C_12_H_24_O_2_)	0.55	11.62
n-Hexadecanoic acid (C_16_H_32_O_2_)	0.36	17.38
3-tert-Butyl-4-hydroxyanisole (C_11_H_16_O_2_)	4.42	11.70
Decanoic acid, 2-methyl (C_11_H_22_O_2_)	2.81	16.72
2-Pyrrolidin-2-yl-1 H-indole (C_12_H_14_N_2_)	12.32	12.58

**Table 3:** Analysis of ingredients in the experimental diet.

Basic ingredients (% total)	Concentration of *W. somnifera* root powder diet (%)			
	0 (control)	1	2.5	4
Fish meal	12	12	12	12
Soybean meal	22	22	22	22
Meat meal	18	18	18	18
Wheat meal	42	41	39.5	38
Sunflower oil	3	3	3	3
Fish oil	1	1	1	1
Vitamin premix^a^	1	1	1	1
Mineral premix^b^	1	1	1	1
*W. somnifera* root powder	0	1	2.5	4
Chemical examination (%)				
Dry matter	88.6	89.1	88.3	88.8
Crude protein	35.8	36	36.1	35.7
Crude fiber	5.4	5.3	5.6	5.5
Crude lipids	8.58	8.55	8.68	8.77
Ash	6.23	6.06	6.13	6.03
Nitrogen free extract^c^	32.59	33.18	31.79	32.79
Energy (kj/g)^d^	17.38	17.49	17.36	17.46

^a^Vitamin premix: A (18,000 IU), B D3 (2,500 U), E (250 mg/kg), K3 (12 mg/kg), B1 (25 mg), B2 (50 mg), B3 (270 mg), B6 (20 mg), B12 (0.06 mg) C (200 mg), folic acid (10 mg), calcium d–pantothenate (50 mg), biotin (1 mg), inositol (120 mg), and choline chloride (2,000 mg). ^b^Mineral premix: iron (75.3 mg), copper (12.2 mg), manganese (206 mg), zinc (85 mg), I (3 mg), selenium (0.350 mg), and cobalt (1 mg). ^c^Nitrogen-free extract (NFE) dry matter – (crude lipid + crude ash + crude protein + crude fiber). ^d^Energy calculated according to 23.6 kJ g^−1^ protein, 39.5 kJ g^−1^ lipid, and 17.0 kJ g^−1^ NFE.

**Table 4:** Effects of various concentrations of *W. somnifera* root powder (WSRP) on the growth and survival rate of *C. carpio*.

Parameters	WSRP0	WSRP1	WSRP2.5	WSRP4	*p* Value
WG (%)	253.52^b^ ± 9.12	262.18^b^ ± 6.81	302.38^a^ ± 3.61	319.13^a^ ± 3.04	0.007
FI (g)	50.71^c^ ± 2.31	50.91^c^ ± 2.09	54.21^b^ ± 2.12	58.51^a^ ± 1.82	0.01
SGR (%)	2.08^b^ ± 0.03	2.12^b^ ± 0.04	2.34^a^ ± 0.03	2.43^a^ ± 0.02	0.02
FCR	1.76^a^ ± 0.05	1.64^b^ ± 0.04	1.42^c^ ± 0.03	1.36^c^ ± 0.03	0.001
PER	1.89^c^ ± 0.05	2.00^b^ ± 0.04	2.24^a^ ± 0.03	2.29^a^ ± 0.03	0.02
SR (%)	100 ± 0.0	100 ± 0.0	100 ± 0.0	100 ± 0.0	—

Means of different superscripts in the same row are different significantly. WG, weight gain; FI, feed intake; SGR, specific growth rate; FCR, feed conversion ratio; PER, protein efficiency ratio; SR, survival rate.

**Table 5:** Effects of various concentrations of *W. somnifera* root powder (WSRP) on hematological indicators of *C. carpio*.

Parameters	WSRP0	WSRP1	WSRP2.5	WSRP4	*p* Value
RBCs (10^6^/mm^3^)	2.35^b^ ± 0.32	2.62^ab^ ± 0.12	2.73^a^ ± 0.10	2.88^a^ ± 0.09	0.03
Hb (gm/dL)	7.29^d^ ± 0.15	7.54^c^ ± 0.12	7.71^b^ ± 0.09	7.94^a^ ± 0.08	0.003
Hct (%)	19.72^c^ ± 2.14	20.83^b^ ± 2.04	21.69^a^ ± 1.83	21.95^a^ ± 1.76	0.01
WBCs (10^3^/mm^3^)	5.42^d^ ± 0.07	5.66^c^ ± 0.05	5.92^b^ ± 0.04	6.12^a^ ± 0.03	0.001
LYM (10^3^/mm^3^)	2.68^c^ ± 0.12	2.98^b^ ± 0.09	3.12^a^ ± 0.07	3.15^a^ ± 0.07	0.002
HET (10^3^/mm^3^)	1.38^c^ ± 0.08	1.45^b^ ± 0.08	1.56^a^ ± 0.07	1.58^a^ ± 0.07	0.02
EOS (10^3^/mm^3^)	0.28^d^ ± 0.007	0.35^c^ ± 0.007	0.41^b^ ± 0.005	0.49^a^ ± 0.004	0.001
MONO (10^3^/mm^3^)	0.58^c^ ± 0.006	0.61^c^ ± 0.003	0.65^b^ ± 0.008	0.71^a^ ± 0.005	0.01

Means of different superscripts in the same row are different significantly. Red blood cells, RBCs; hemoglobin, Hb; hematocrit, Hct; white blood cells, WBCs; lymphocytes, LYM; heterophils, HET; eosinophils, EOS; monocytes, MONO.

**Table 6:** Effects of various concentrations of *W. somnifera* root powder (WSRP) on biochemical indices of *C. carpio*.

Parameters	WSRP0	WSRP1	WSRP2.5	WSRP4	*p* Value
ALB (g/dL)	2.28^d^ ± 0.09	2.59^c^ ± 0.06	2.84^b^ ± 0.05	3.04^a^ ± 0.04	0.006
TP (g/dL)	5.18^c^ ± 0.18	6.06^b^ ± 0.12	6.48^a^ ± 0.13	6.84^a^ ± 0.08	0.002
GLOB (g/dL)	2.86^c^ ± 0.16	3.42^b^ ± 0.13	3.58^ab^ ± 0.09	3.75^a^ ± 0.06	0.01
GLU (mg/dL)	73.71^a^ ± 2.41	71.05^a^ ± 2.06	65.74^b^ ± 1.63	60.61^c^ ± 1.22	0.003
CORT (ng/L)	51.31^a^ ± 1.82	49.33^a^ ± 1.27	44.52^b^ ± 1.05	41.46^c^ ± 0.87	0.002

Means of different superscripts in the same row are different significantly. Albumin, ALB; total proteins, TP; globulin, GLOB; glucose, GLU; cortisol, CORT.

**Table 7:** Effects of various concentrations of *W. somnifera* root powder (WSRP) on antioxidant status of *C. carpio*.

Parameters	WSRP0	WSRP1	WSRP2.5	WSRP4	*p* Value
TAC (mM/L)	1.58^c^ ± 0.07	1.69^c^ ± 0.06	2.06^b^ ± 0.12	3.15^a^ ± 0.17	0.003
CAT (U/L)	66.92^d^ ± 2.74	74.63^c^ ± 2.96	86.62^b^ ± 2.46	93.68^a^ ± 3.01	0.002
SOD (U/mL)	6.05^c^ ± 0.83	7.09^c^ ± 0.63	9.21^b^ ± 0.37	12.09^a^ ± 0.84	0.002
GSH (*µ*mol/mL)	10.83^c^ ± 0.79	12.23^c^ ± 0.69	14.88^b^ ± 0.92	17.32^a^ ± 0.97	0.001

Means of different superscripts in the same row are different significantly. Total antioxidant capacity, TAC; catalase, CAT; superoxide dismutase, SOD; reduced glutathione, GSH.

## Data Availability

The data that support the findings of this study are available from the corresponding author upon reasonable request.
